# Tuning of Photocatalytic and Piezophotocatalytic Activity of Bi_3_TiNbO_9_ via Synthesis-Controlled Surface Defect Engineering

**DOI:** 10.3390/molecules30204136

**Published:** 2025-10-20

**Authors:** Farid F. Orudzhev, Asiyat G. Magomedova, Sergei A. Kurnosenko, Vladislav E. Beklemyshev, Wei Li, Chuanyi Wang, Irina A. Zvereva

**Affiliations:** 1 Smart Materials Laboratory, Dagestan State University, St. M. Gadjieva 43-a, Dagestan Republic, Makhachkala 367000, Russia; asiyat_magomedova1996@mail.ru; 2 Geothermal and Renewal Energy Institute of the Russian Academy of Sciences, Ave. 39-a, Dagestan Republic, Makhachkala 367030, Russia; 3 Department of Chemical Thermodynamics and Kinetics, Saint Petersburg State University, 7/9 Universitetskaya nab., St. Petersburg 199034, Russia; s.kurnosenko@spbu.ru (S.A.K.); st117562@student.spbu.ru (V.E.B.); 4 School of Chemistry and Chemical Engineering, Shaanxi University of Science and Technology, Xi’an 710021, China; liweihg@sust.edu.cn; 5 School of Environmental Science and Engineering, Shaanxi University of Science and Technology, Xi’an 710021, China; wangchuanyi@sust.edu.cn

**Keywords:** Bi_3_TiNbO_9_, Aurivillius phases, perovskite-type layered oxide, piezophotocatalysis, piezocatalysis, photocatalysis, methylene blue degradation

## Abstract

In this work, we investigate advanced photocatalyst Bi_3_TiNbO_9_ as promising piezophotocatalyst in terms of the effect of synthesis methods on the surface chemistry, structure, and catalytic performance in process of contaminant removal. Samples were prepared via solid-state reaction (BTNO-900) and molten salt synthesis (BTNO-800), leading to distinct morphologies and defect distributions. SEM imaging revealed that BTNO-900 consists of agglomerated, irregular particles, while BTNO-800 exhibits well-faceted, plate-like grains. Nitrogen adsorption analysis showed that the molten-synthesized sample possesses a significantly higher specific surface area (5.9 m^2^/g vs. 1.4 m^2^/g) and slightly larger average pore diameter (2.8 nm vs. 2.6 nm). High-resolution XPS revealed systematic shifts in binding energies for Bi 4f, Ti 2p, Nb 3d, and O 1s peaks in BTNO-900, accompanied by a higher content of adsorbed oxygen species (57% vs. 7.2%), indicating an increased concentration of oxygen vacancies and surface hydroxylation due to the solid-state synthesis route. Catalytic testing demonstrated that BTNO exhibits enhanced piezocatalytic efficiency of Methylene Blue degradation (~78% for both samples), whereas BTNO-800 shows significantly reduced photocatalytic activity (45.6%) compared to BTNO-900 (84.1%), suggesting recombination effects dominate in the more defective material. Synergism of light and mechanical stress results in piezophotocatalytic degradation for both samples (92.4% and 93.4%, relatively). These findings confirm that synthesis-controlled defect engineering is a key parameter for optimizing the photocatalytic behavior of Bi_3_TiNbO_9_-based layered oxides and crucial role of its piezocatalytic activity.

## 1. Introduction

In recent decades, environmental protection and the prevention of water pollution have become major global challenges. Among the most hazardous sources of water contamination are wastewater containing organic dyes, which are discharged as a result of activities in various industrial sectors such as textile, pharmaceutical, printing, and paper industries. When these organic dyes enter aquatic environments, they can cause serious ecological consequences, including a reduction in dissolved oxygen levels, blockage of light penetration, and toxic effects on both flora and fauna. As a result, there is an urgent need to develop effective methods for wastewater treatment prior to its discharge into the environment [1,2].

In response to this challenge, researchers worldwide are actively engaged in the search for and development of technologies aimed at the effective removal of persistent organic pollutants from aqueous media. In this field, special attention is paid to so-called advanced oxidation processes (AOPs), which encompass a series of chemical reactions based on the generation of reactive oxygen species (ROS) [3,4,5,6,7]. These reactive species are capable of degrading recalcitrant organic compounds into simpler and safer products, such as carbon dioxide, water, and mineralized salts. AOPs are already widely used in various wastewater treatment applications, demonstrating high efficiency in the decomposition of dyes and other toxic substances.

One of the promising directions within AOPs is the use of photocatalysis and piezocatalysis for the degradation of organic pollutants. Photocatalysis is based on the use of light to activate photocatalysts, which initiate oxidative processes leading to the breakdown of organic molecules. In this process, light serves as an energy source that generates active electrons and holes necessary for redox reactions [8,9]. Photocatalysis is widely applied in wastewater treatment; however, its efficiency is often limited by illumination conditions and insufficient charge carrier separation rates [10,11,12,13].

Piezocatalysis is a relatively new approach that utilizes mechanical stress to activate catalytic processes. Unlike photocatalysis, piezocatalysis does not require light, making it more versatile in its applications. When mechanical stress is applied to piezoelectric materials, an internal electric field is generated within their structure, which facilitates the separation and migration of charge carriers. This field accelerates oxidation and reduction processes, thereby enabling the efficient degradation of pollutants without significant energy consumption [14,15,16,17]. Piezocatalysis combines the advantages of mechanochemistry and catalysis, offering an environmentally friendly solution for wastewater purification.

Combining photocatalysis and piezocatalysis within a single material opens new horizons for enhancing the efficiency of these processes [18,19]. Materials that possess both piezoelectric and photocatalytic properties can simultaneously utilize light energy and mechanical stress to activate catalytic processes, enabling a significant increase in the rate of pollutant degradation [20,21,22,23,24].

In this context, layered bismuth-based materials, such as those belonging to the Aurivillius phase family, have attracted particular attention. These materials have a unique crystal structure consisting of alternating [Bi_2_O_2_]^2+^ layers and anionic groups, which provide favorable conditions for the generation and transport of charges [25]. Moreover, the Aurivillius structure allows for easy tuning of the band gap width and modification of lattice defects, making these materials particularly promising for catalytic applications [14]. Aurivillius-type materials possess several advantages that make them ideal candidates for use as piezophotocatalysts. Firstly, their unique layered structure enables efficient control of charge carrier transport, which is especially important for inhibiting the recombination of electrons and holes—a factor limiting the efficiency of many traditional photocatalysts. Secondly, the presence of piezoelectric properties in such materials opens up the possibility of using mechanical stress for additional activation of catalytic processes. The internal electric field generated in piezoelectric materials under deformation facilitates charge carrier separation, thereby increasing the number of reactive species involved in the oxidation of organic pollutants [26].

The synthesis and investigation of Aurivillius family materials have become the focus of active scientific interest. Bismuth-containing materials not only demonstrate high efficiency in the degradation of pollutants but also allow for the use of ultrasonic treatment to enhance catalytic activity [27].

In [28], a new structured Sillen–Aurivillius material, Bi_4_W_0.5_Ti_0.5_O_8_Cl, was synthesized and utilized for piezophotocatalytic oxygen activation. Bi_4_W_0.5_Ti_0.5_O_8_Cl exhibited excellent efficiency in oxygen activation via piezophotocatalysis. The maximum H_2_O_2_ evolution rate reached 530.4 μmol·h^−1^·g^−1^. In the study by Hao Kai et al., Bi_4_O_5_Br_2_, which utilizes oxygen vacancies with appropriate concentration and thickness of several atomic layers, demonstrated impressive H_2_O_2_ evolution rates (620 μmol·g^−1^·h^−1^ in pure water, 2700 μmol·g^−1^·h^−1^ in a sacrificial system) [29]. In [30], BaBi_4_TiNbO_11_Cl nanoplatelets used for piezophotocatalytic degradation of RhB exhibited 100% efficiency within 30 min. Xie et al. reported that the photocatalytic properties of Bi_4_Ti_3_O_12_ nanoplatelets were significantly enhanced due to the introduction of the piezophototronic effect [31]. Bi_4_Ti_3_O_12_ nanoplatelets synthesized by a molten salt method demonstrated that the piezophotocatalytic degradation rate constant k for RhB reached 0.1414 min^−1^, which is 5.6 times greater than the piezocatalytic k value and 2.1 times greater than the photocatalytic k value [31,32].

The use of Bi_3_TiNbO_9_ as a piezophotocatalyst for water purification from organic dyes is of considerable interest from the perspective of sustainable and environmentally friendly solutions to pollution. In [33], Bi_3_TiNbO_9_ nanosheets were synthesized via a one-step hydrothermal method using NaOH as a mineralizer. Their piezocatalytic and piezophotocatalytic degradation characteristics were investigated. It was found that the BTNO degradation rate constant under L + U was 0.03889 min^−1^, which is 3.19 and 1.44 times higher than those under ultrasound (0.01219 min^−1^) and visible light (0.02698 min^−1^), respectively. Similarly, the BTNO k value under S + U (0.05212 min^−1^) was 1.55 times higher than under simulated sunlight (0.03368 min^−1^). These results confirm the synergistic effect of light irradiation and ultrasonic vibration, attributable to the crucial role of piezoelectric polarization in accelerating the separation and transfer of photogenerated charges.

The aim of this study is to reveal how synthesis-controlled defect engineering affects the photocatalytic, piezocatalytic, and piezophotocatalytic activity of Bi_3_TiNbO_9_. For this purpose, two samples obtained via ceramic solid-state synthesis and molten-salt method were systematically compared.

## 2. Results and Discussion

### 2.1. Structure and Texture Properties

**Figure 1:**
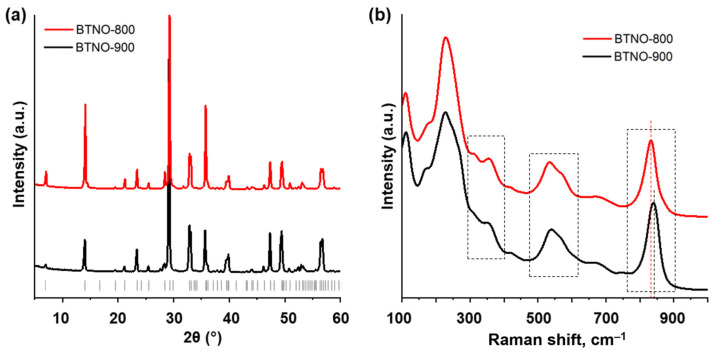
Powder XRD patterns (**a**) and Raman spectra (**b**) of the ceramic BTNO-900 and melt-synthesized BTNO-800 samples of Bi_3_TiNbO_9_. The dashed rectangles highlight specific regions of the spectra being discussed in the text.

The Bi_3_TiNbO_9_ materials prepared by two different approaches were primarily identified by the means of powder XRD analysis (Figure 1a). Both ceramic (BTNO-900) and melt-synthesized (BTNO-800) oxides represent well crystallized samples giving distinct diffraction maxima and not containing significant amounts of crystalline impurity phases. XRD patterns of the materials obtained are consistent with those from the ICDD database and best match the card № 01-072-7962 corresponding to orthorhombic space group A21am. typical for ferroelectric Aurivillius-phase oxide. The lattice parameters of ceramic and melt-synthesized Bi_3_TiNbO_9_ (Table 1) are close in *a* and *b* parameters and slightly different in *c* parameter. It is general that *c* parameter stronger depends on method of synthesis as it could be visible in comparison with data obtained for single crystal [34] and sample synthesized by hydrothermal method [35].

Raman spectra of the ceramic and melt-synthesized Bi_3_TiNbO_9_ (Figure 1b) are generally consistent with those presented in the available literature and show clearly distinguishable characteristic peaks corresponding to the O–M–O bending (300–400 cm^−1^), opposing excursions of the external apical oxygens in the MO_6_ octahedra (500–600 cm^−1^) and symmetric stretching of the axial M–O bonds directed towards the interlayer space (800–900 cm^−1^) (M = Ti, Nb) [35]. At the same time, the vibrational modes of the two Bi_3_TiNbO_9_ samples are not completely identical. Particularly, the aforementioned M–O stretching in the ceramic material (840 cm^−1^) is shifted towards greater frequencies as compared to that of the melt-synthesized one (832 cm^−1^), which might indicate a somewhat higher distortion of the octahedra (elongation) in sample’s lattice [34] that results in the increasing of the c parameter (Table 1).

**Figure 2:**
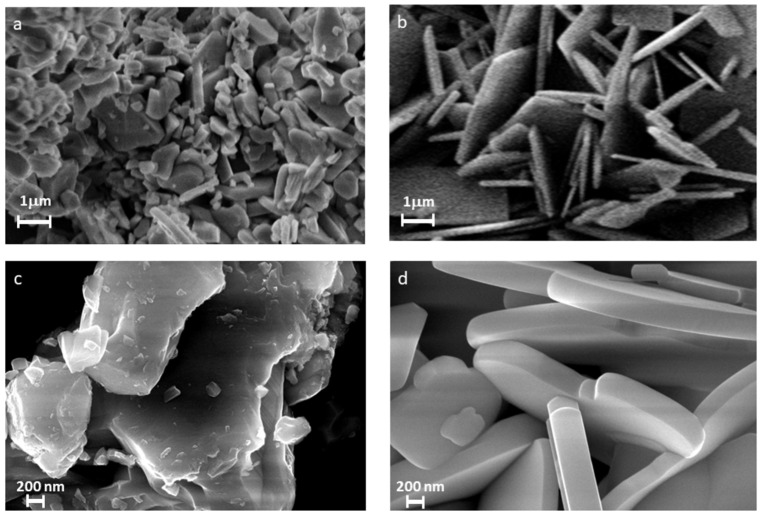
SEM images of BTNO-900 (**a**,**c**) and BTNO-800 samples (**b**,**d**).

Figure 2 presents the SEM micrographs of Bi_3_TiNbO_9_ samples synthesized via solid-state reaction (Figure 2a) and molten salt method (Figure 2b).

The ceramic sample (BTNO-900, Figure 2a) exhibits an aggregated microstructure composed of irregularly shaped particles, showing pronounced agglomeration and limited growth of individual crystallites. Such morphology is indicative of a low degree of texturing and poor control over grain growth, which is typical of conventional solid-state synthesis. In contrast, the sample obtained via the molten salt route (BTNO-800, Figure 2b) displays well-defined, plate-like grains with pronounced facets and moderate agglomeration. At higher magnification (Figure 2c,d), these differences become even more pronounced. The BTNO-900 sample (Figure 2c) shows coarse particles with uneven, defect-rich surfaces, whereas the BTNO-800 sample (Figure 2d) exhibits uniform and clean faceted grains with sharp edges and well-developed planes, reflecting a more ordered layered structure and enhanced crystal growth control. This suggests a more oriented grain growth and a well-ordered layered structure characteristic of Aurivillius-type phases. The observed morphology is consistent with the XPS results, which reveal that BTNO-800 possesses a more structurally ordered surface enriched in lattice oxygen, with minimal contributions from surface defects and adsorbed species.

The elemental composition and distribution of the main components in Bi_3_TiNbO_9_ were further confirmed by EDX analysis (see Table S1 and Figures S1 and S2 in the Supplementary Materials). The spectra reveal the presence of Bi, Ti, Nb, and O as the major elements, in agreement with the expected stoichiometry. Trace signals of Na, K, and Cl (<0.5 at.%) were detected only for the BTNO-800 sample, which can be attributed to residual precursor salts or adsorbed ionic species. The uniform distribution of the main elements supports the formation of a single-phase Bi3TiNbO9 structure.

These morphological distinctions are further supported by nitrogen adsorption measurements (Table 2 see also Figure S3 in the Supplementary Materials). The melt-synthesized BTNO-800 sample demonstrates a significantly higher specific surface area (5.9 m^2^/g) compared to the ceramic BTNO-900 (1.4 m^2^/g), which correlates with its pronounced texturing observed in the SEM images. The average pore diameters for both samples are similar (2.8 nm and 2.6 nm, respectively), indicating a retained mesoporous structure in both cases. However, the lower total pore volume observed for BTNO-800 (0.005 cm^3^/g) relative to BTNO-900 (0.008 cm^3^/g) suggests denser packing and lower overall porosity in the melt-synthesized material.

Taken together, the morphological and textural differences revealed by SEM and nitrogen physisorption analyses clearly demonstrate the substantial impact of the synthesis route on the surface architecture of Bi_3_TiNbO_9_. The more ordered morphology and increased specific surface area of BTNO-800 indicate a greater degree of structural integrity, which likely influences the distribution and nature of surface-active centers and oxygen-containing species.

### 2.2. Surface Chemistry

To further elucidate the differences in surface chemistry between the Bi_3_TiNbO_9_ samples synthesized by molten salt (BTNO-800) and solid-state reaction (BTNO-900), high-resolution X-ray photoelectron spectroscopy (XPS) analysis was conducted.

**Figure 3:**
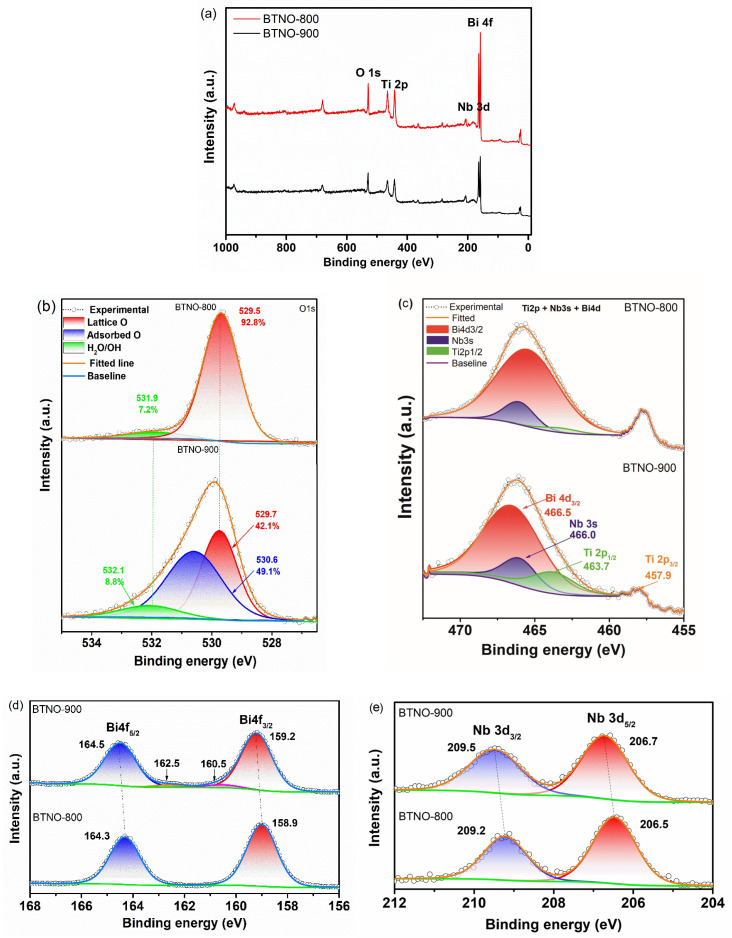
XPS of the BTNO-900 and BTNO-800: wide spectra (**a**) O 1s (**b**), Ti 2p + Bi 4d + Nb 3s (**c**), Bi 4f (**d**), and Nb 3d (**e**) regions. In subfigures (**d**,**e**), the different colors are used solely for visual clarity and represent the same element type.

The survey XPS spectrum (Figure 3a) displays well-defined peaks corresponding to the main constituent elements of Bi_3_TiNbO_9_, namely O 1s, Ti 2p, Nb 3d, and Bi 4f. No significant contamination signals were detected, confirming the chemical purity of the sample surfaces. However, the relative intensities of specific peaks vary depending on the synthesis route. In the melt-synthesized sample (BTNO-800), the Bi 4f peak is notably more intense, whereas the O 1s signal is reduced, which may indicate differences in the density of oxygen-related surface species and the coordination environment of cations.

High-resolution O 1s spectra (Figure 3b) reveal pronounced differences in oxygen coordination. For BTNO-800, the O 1s signal is dominated by lattice oxygen, accounting for 92.8% of the total oxygen species at a binding energy of 529.5 eV, with only a minor contribution (7.2%) from adsorbed oxygen species. In contrast, BTNO-900 exhibits a shift in the O 1s binding energy to 529.7 eV, accompanied by a substantial increase in the proportion of surface-adsorbed oxygen and hydroxyl groups—collectively exceeding 57%. These changes are indicative of a significantly higher concentration of oxygen vacancies and surface defects, most likely resulting from limited ion diffusion and uneven crystallite growth during solid-state synthesis.

The high-resolution XPS spectra of Ti 2p for the BTNO samples (Figure 3c) exhibit Ti 2p_3/2_ peaks centered at 457.9 eV, which confirms the presence of Ti in the +4 oxidation state, in agreement with previous reports [33]. It should be noted that the spectral region from 460 to 472 eV contains overlapping contributions from Ti 2p_1/2_, Nb 3s, and Bi 4d_3/2_ levels. Accordingly, the composite peak was deconvoluted into three distinct components. For BTNO-900, these components were identified at 463.7 eV (Ti 2p_1/2_), 466.0 eV (Nb 3s), and 466.5 eV (Bi 4d_3/2_). The observed shifts in these peaks to higher binding energies in BTNO-900 may be attributed to changes in the local electronic environment of the cations and have previously been associated with enhanced local polarization effects and coordination distortions in defect-rich Aurivillius-type structures [35].

The Bi 4f spectra (Figure 3d) of BTNO-800 exhibit well-defined peaks at 158.9 eV and 164.3 eV, corresponding to Bi 4f_7/2_ and Bi 4f_5/2_, respectively. These binding energies are characteristic of Bi^3+^ in a symmetric coordination environment. In contrast, BTNO-900 shows a noticeable shift in the Bi 4f peaks toward higher binding energies (159.2 eV and 164.5 eV), accompanied by the appearance of shoulder components, indicating a modified local coordination of bismuth and the influence of polarizable structural defects, as previously reported for related Aurivillius-type phases [38]. The shift in peak positions without a change in oxidation state suggests a redistribution of electron density around Bi ions, likely due to an increased concentration of oxygen vacancies.

In the Nb 3d spectra (Figure 3e), BTNO-800 displays characteristic Nb 3d_5/2_ and Nb 3d_3/2_ peaks at 206.5 eV and 209.2 eV, respectively, consistent with the Nb^5+^ oxidation state. For BTNO-900, these peaks are shifted to 206.7 eV and 209.5 eV, which may be attributed to changes in the local coordination environment and electronic structure, driven by a higher density of oxygen vacancies and surface defects induced by the solid-state synthesis route.

These findings suggest that solid-state synthesis leads to a more defect-rich surface with elevated concentrations of oxygen vacancies and chemisorbed oxygen species. Such defects can facilitate the formation of reactive oxygen species (ROS), contributing to enhanced piezocatalytic activity. However, their excessive presence may also promote charge carrier recombination, thereby reducing the efficiency of photocatalytic processes.

### 2.3. Optical Properties

**Figure 4:**
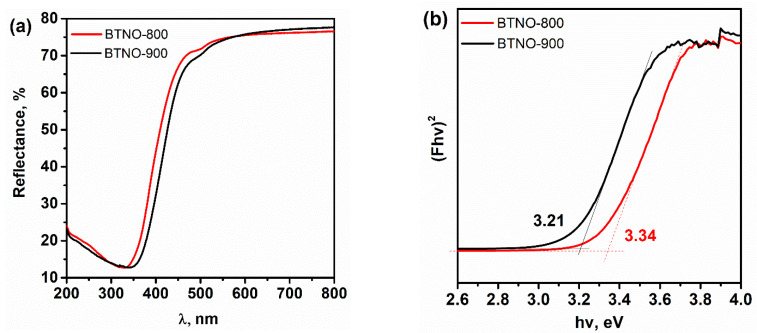
DRS (**a**) and Kubelka–Munk plots (**b**) for the BTNO-800 and BTNO-900.

To investigate the optical properties BTNO-900 and BTNO-800 Bi_3_TiNbO_9_ samples, diffuse reflectance spectroscopy (DRS) combined with the Kubelka–Munk transformation was employed (Figure 4a,b). Both materials exhibit a pronounced absorption edge in the near-UV region, associated with interband electronic transitions. Notably, the absorption edge of the ceramic BTNO-900 sample is red-shifted toward longer wavelengths (λ_max_ = 386 nm) compared to BTNO-800 (λ_max_ = 371 nm), indicating a reduction in the effective optical band gap for BTNO-900 (see Table 3). The obtained values of the bandgap energy are in good agreement with the data available in the literature: 3.31–3.33 eV for synthesis in molten salts and 3.27 eV for solidstate synthesis [33,36,37,39].

**Figure 5:**
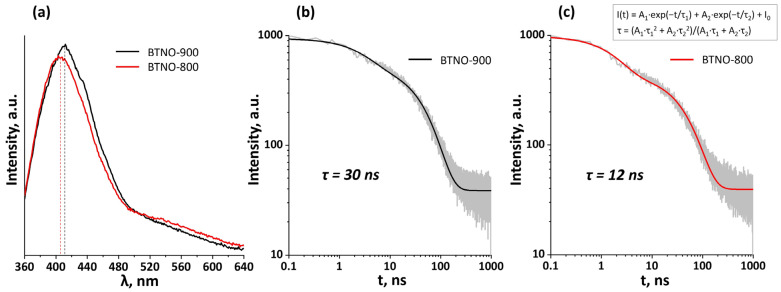
Photoluminescence spectra measured with 340 nm excitation (**a**) and decay graphs for the BTNO-900 (**b**) and BTNO-800 (**c**).

The optical band gap energies (E_g_) were determined by extrapolating the linear portion of the (F(R)hν)^2^–hν plots to the energy axis. For BTNO-900, Eg was found to be 3.21 eV, whereas for BTNO-800 it was 3.34 eV. It should be emphasized that the reduction in the optical band gap observed for BTNO-900 is most likely not associated with a fundamental shift in the conduction or valence band edges, but rather with the formation of defect-induced sub-band states within the forbidden gap, caused by the higher concentration of oxygen vacancies and surface states revealed by XPS analysis. These defect-related energy levels facilitate optical transitions at lower photon energies, leading to the red shift in the absorption edge. The apparent narrowing of the band gap and the increased density of defect states characteristic of BTNO-900 are expected to directly influence the recombination dynamics of photogenerated charge carriers. To verify this assumption, photoluminescence (PL) and time-resolved PL (TRPL) studies were performed (Figure 5) to evaluate the recombination kinetics of electron–hole pairs and correlate them with the morphological and defect-chemical differences between the samples.

Upon excitation at 340 nm, both samples exhibit pronounced emission bands in the 405–410 nm region, consistent with the position of the optical absorption edge (see Table 3). However, the PL intensity for BTNO-800 is significantly lower than that for BTNO-900. Analysis of the photoluminescence decay kinetics revealed that the average lifetime of photogenerated carriers in BTNO-900 (τ = 30 ns) is approximately 2.5 times longer than in BTNO-800 (τ = 12 ns). The longer carrier lifetime in BTNO-900 correlates with its higher concentration of oxygen vacancies and surface hydroxyl groups, which facilitate spatial separation of electrons and holes via local internal electric fields.

At first glance, the combination of high PL intensity and prolonged lifetime in BTNO-900 might appear contradictory, since strong luminescence is typically associated with fast radiative recombination and thus shorter carrier lifetimes. However, a more detailed analysis of the recombination processes shows that such behavior is characteristic of systems in which defects and internal electric fields simultaneously retard total recombination—leading to longer τ—while suppressing nonradiative channels, thus ensuring a high quantum efficiency of emission. In BTNO-900, the high concentration of oxygen vacancies and surface hydroxyl groups revealed by XPS analysis creates localized states within the band gap and internal electric fields that hinder the rapid spatial annihilation of electron–hole pairs. These fields enable spatial separation of carriers, reducing the probability of nonradiative recombination and increasing the fraction of slower radiative processes, which manifests as both higher PL intensity and longer carrier lifetimes. In contrast, in BTNO-800, where the concentration of defects and internal electric fields is lower, carriers undergo rapid recombination, with a substantial fraction lost through nonradiative pathways, leading to both reduced lifetimes and lower PL intensity.

Thus, the combination of enhanced PL intensity and longer carrier lifetimes in BTNO-900 reflects the key role of defect-induced internal fields and carrier localization in suppressing nonradiative recombination and enhancing radiative efficiency. These processes enable prolonged survival of separated electron–hole pairs, providing a mechanistic basis for the enhanced photocatalytic activity of the ceramic sample, which will be discussed in the following section.

### 2.4. Catalytic Activity

**Figure 6:**
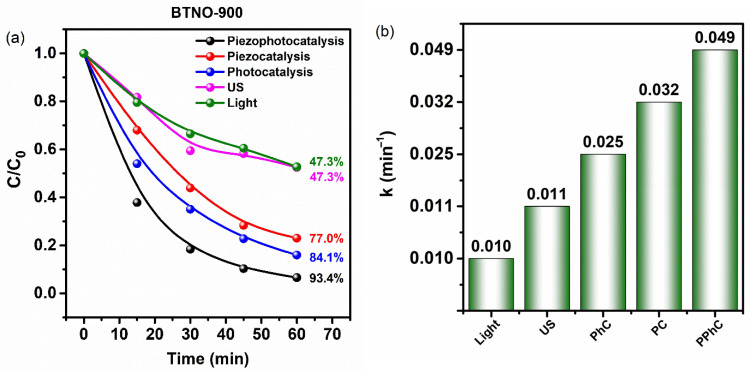

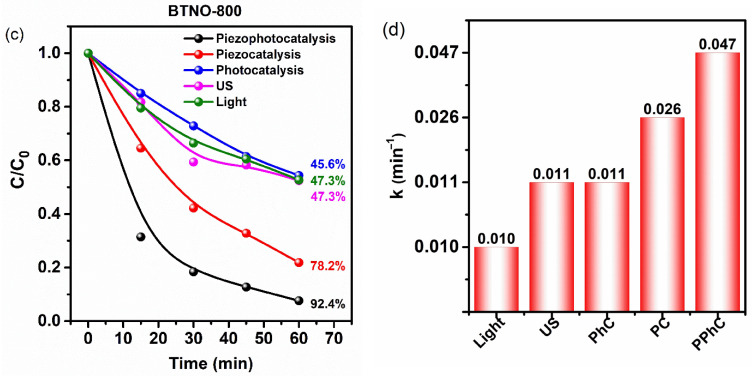
Photocatalytic, piezocatalytic, and piezophotocatalytic activities of BTNO-900 (**a**,**b**) and BTNO-800 (**c**,**d**). Panels (**a**,**c**) show the degradation curves of MB under different conditions (light, ultrasound, photocatalysis, piezocatalysis, and piezophotocatalysis). Panels (**b**,**d**) present the corresponding apparent first-order rate constants (k).

The catalytic activity of the samples was investigated via the degradation of MB dye. In order to accurately assess the effect of the catalysts on MB decomposition, it was first necessary to study the impact of ultrasound (US) and light separately, in the absence of catalysts. The results are presented in Figure 6. It is evident that sonolysis of the MB solution resulted in approximately 47% degradation within 60 min. This result is attributed to the cavitation effect, as it is well known that rapid pressure fluctuations in water during periodic compression and expansion lead to the formation and growth of voids or microbubbles [7]. Both theoretical and experimental studies have demonstrated that adiabatic collapse of microbubbles creates local regions with high pressure (~1000 atm) and temperature (~5000 K). These conditions provide sufficient energy for the dissociation of water molecules, especially at the gas–liquid interface, resulting in the formation of highly reactive ·OH and ·H radicals [40]. In a similar control experiment with only light exposure (photolysis) (Figure 6a), the degree of MB degradation after 60 min was also 47%. To eliminate the influence of dye adsorption on the assessment of photo-, piezo-, and piezophotocatalytic activities, prior to each experiment, the samples were stirred in MB solution in the dark until adsorption–desorption equilibrium was reached. According to the results, equilibrium was achieved after approximately 30 min, with a decrease in MB concentration of about 30% for both samples. The samples exhibited different activities in the photocatalytic experiments. For the BTNO-900 the MB concentration decreased by approximately 84%, indicating good photocatalytic activity, whereas for the BTNO-800, the result did not exceed that of photolysis (Figure 6c), at about 45%. This is likely due to rapid recombination of charge carriers, as demonstrated by the time-resolved photoluminescence experiments. The charge carrier lifetime for BTNO-800 was 2.5 times shorter than for BTNO-900: 12 ns and 30 ns.

Subsequently, the piezocatalytic activity of the samples was studied using an ultrasonic bath as a source of mechanical stimulation. The results of MB degradation under piezocatalytic conditions showed that both samples exhibited similar catalytic activity, with a reduction in methylene blue concentration of about 78%. The activity observed under ultrasonic irradiation can be interpreted based on mechanisms described in [41], with the following being the most probable:

1. Indirect sonolysis and sonocatalysis: These processes occur due to the cavitational pyrolysis of water, where hydroxyl radical generation is caused by the formation and collapse of cavitation bubbles. The contribution of sonolysis is well-established and can be controlled. According to sonocatalytic theory, enhancement of the effect upon addition of nanoparticles is attributed to their ability to either enhance or suppress cavitation, depending on their morphology and size. However, despite differences in size and morphology between the studied nanoparticles, their piezocatalytic activities were comparable. Therefore, this mechanism does not explain the observed results.
2. Photocatalytic and thermocatalytic mechanisms (PCM, TCM): According to these theories, sonoluminescence or local temperature increase during cavitation can initiate the generation of electron-hole pairs in the semiconductor catalyst, enhancing the catalytic process. However, since the samples exhibit different photocatalytic activities, these mechanisms also cannot explain the identical results obtained under piezocatalytic conditions.

Thus, the most probable explanation for the observed experimental data is the presence of the piezoelectric effect in the studied materials. According to this hypothesis, ultrasonic irradiation induces piezoelectric polarization and an internal electric field at the particle surface, which promote charge separation and interfacial charge transfer, thereby initiating a cascade of redox reactions.

It is known that materials exhibiting both photo- and piezocatalytic activities are capable of demonstrating the piezophototronic effect, resulting in enhanced catalytic activity under simultaneous action of both effects. To verify this, a piezophotocatalytic experiment was conducted. As shown in Figure 6a,c, the degree of MB degradation after 60 min under these conditions reached approximately 93% for both samples, indicating a pronounced synergistic effect. Particularly noteworthy is the result for the BTNO-800, which initially did not exhibit significant photocatalytic activity. The substantial increase in reaction rate under piezophotocatalytic conditions, 4.3 and 1.8 times higher compared to photocatalysis and piezocatalysis, respectively, can be attributed to the generation of an electric field via the piezoelectric effect. This electric field promotes spatial separation of electron-hole pairs, leading to increased charge carrier lifetimes and, consequently, higher catalytic efficiency. The reaction rate constants calculated using the pseudo-first-order Langmuir-Hinshelwood model from the slope of –ln(C/C_0_) versus time (Figure S4), support these conclusions and are presented in the diagrams in Figure 6b,d. Under ultrasound irradiation without catalyst, the MB degradation rate constant was relatively low at k = 0.012 min^−1^, while in the presence of the catalyst, k = 0.032 min^−1^, indicating that the catalyst is an integral component of the piezocatalytic reaction. The rate of MB degradation via piezocatalysis (k = 0.032 min^−1^) exceeded that of sonolysis by 2.6 times for BTNO-900 and by 2.4 times for BTNO-800.

Under piezophotocatalytic conditions, the rate constants were k = 0.049 min^−1^ for BTNO-900 and k = 0.047 min^−1^ for BTNO-800, nearly four times higher than the rates for sonolysis and photolysis, thus confirming the synergistic effect of the combined piezo- and photocatalytic processes.

In order to confirm the validity of the adopted kinetic model, pseudo-first-order (Langmuir–Hinshelwood type) and second-order equations were tested for all degradation modes using BTNO-800 as an example. A comparison of the correlation coefficients (R^2^) and rate constants (Table S2, Supplementary Materials) demonstrates that the pseudo-first-order model yields R^2^ values that are marginally higher or equivalent to those of the second-order model (0.97–0.99), thereby substantiating its adequacy within the confines of our experimental setup. Consequently, the pseudo-first-order kinetics was choosen for the purpose of further analysis and discussion.

The morphological and compositional stability of the BTNO-800 catalyst after the piezophotocatalytic process was further verified by SEM and EDX analyses. As shown in the Supplementary Materials (Figure S5), the Bi_3_TiNbO_9_ particles retain their original plate-like morphology and chemical composition after repeated catalytic cycles. The impurity peaks of Na, K, and Cl observed in the pristine BTNO-800 sample completely disappear after catalysis, indicating a surface self-cleaning effect and high structural stability of the material under piezophotocatalytic conditions.

### 2.5. Reaction Mechanism/Trapping Experiments

To elucidate the piezocatalytic mechanism and provide a deeper analysis of MB degradation processes, trapping experiments were conducted to identify the key reactive species involved in the piezophotocatalytic degradation using Bi_3_TiNbO_9_ under ultrasonic irradiation. The experimental methodology was similar to the procedure used for the evaluation of piezocatalytic activity, except for the addition of specific scavengers to selectively inhibit certain reactive species.

**Figure 7:**
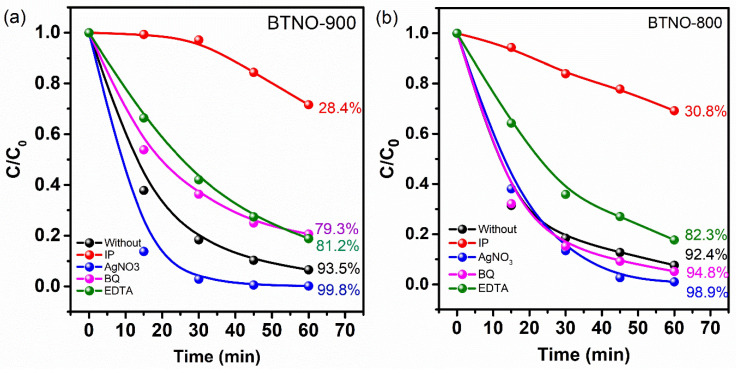
Trapping experiments for BTNO-900 (**a**) and BTNO-800 (**b**).

The following scavengers were used: isopropyl alcohol (IPA) to capture hydroxyl radicals (·OH), benzoquinone (BQ) to trap superoxide radicals (·O_2_^−^), disodium ethylenediaminetetraacetate (EDTA-2Na) as a hole (h^+^) scavenger, and silver nitrate (AgNO_3_) as an electron scavenger. The results are presented in Figure 7.

After the introduction of isopropyl alcohol (IPA), used as a scavenger for hydroxyl radicals (·OH), the degradation efficiency of MB was significantly reduced, reaching 28.4% for the BTNO-900 and 30.8% for the BTNO-800. This indicates that ·OH are the dominant reactive species in the piezophotocatalytic process.

The effect of adding benzoquinone (BQ), used as a scavenger for superoxide radicals (·O_2_^−^), was ambiguous. For the BTNO-900, inhibition of the degradation process to 73.3% was observed, indicating a significant role of superoxide radicals in the degradation mechanism. In contrast, for the BTNO-800 sample, the addition of BQ resulted in only a slight acceleration of the reaction, suggesting either the absence of superoxide radicals (·O_2_^−^) or their negligible formation in this case. The addition of disodium ethylenediaminetetraacetate (EDTA-2Na), used as a hole (h^+^) scavenger, led to a similar inhibition of the process for both catalysts, reducing the degradation efficiency to 81–82%. This highlights the importance of holes (h^+^) as active species in the piezophotocatalytic reaction for both samples. Thus, the results of radical trapping experiments demonstrate the differing roles of reactive species depending on the synthesis conditions and properties of the catalysts.

The addition of silver nitrate (AgNO_3_), which acts as an electron scavenger, led to an acceleration of MB degradation for both catalysts. This phenomenon can be explained by the occurrence of redox reactions, which enhance catalytic activity. Silver nitrate (Ag^+^) captures electrons, thereby preventing electron-hole recombination and increasing the concentration of active holes. The deposition of metallic silver (Ag) on the catalyst surface may further catalyze oxidation reactions.

Based on the results of the trapping experiments, a reaction mechanism for the piezophotocatalytic degradation occurring on the surface of Bi_3_TiNbO_9_ under ultrasonic vibration and light irradiation is proposed. The main processes start with the photo-generation of electron–hole pairs under illumination:

Bi_3_TiNbO_9_ + hν → e^−^ + h^+^

Concurrently, ultrasonic deformation induces a piezoelectric field that enhances spatial separation and interfacial transport of these carriers (piezo-phototronic effect), thereby facilitating the formation of reactive species.

Holes (h^+^) interact with water molecules or hydroxide ions to generate hydroxyl radicals (·OH), which are powerful oxidizing agents that play a key role in the degradation of organic pollutants such as MB:

h^+^ + H_2_O → ·OH + H^+^

h^+^ + OH^−^ → ·OH

Superoxide radicals (·O_2_^−^) are formed as a result of the reaction between electrons (e^−^) and dissolved oxygen, also contributing to redox processes. The resulting superoxide radicals can undergo further reactions with protons or water to form hydroperoxyl radicals (·HO_2_) and hydrogen peroxide (H_2_O_2_), which subsequently decompose into hydroxyl radicals:

e^−^ + O_2_ → ·O_2_^−^ (For the ceramic sample, the amount of adsorbed oxygen is higher, and consequently, ·O_2_^−^ is also higher.)

O_2_^−^ + H^+^ → ·HO_2_

2·HO_2_ → H_2_O_2_ + O_2_

The addition of silver nitrate (AgNO_3_) as an electron scavenger leads to reaction acceleration by preventing electron-hole recombination. Ag^+^ captures electrons and is reduced to metallic silver (Ag), which can further catalyze oxidation reactions. As a result, the concentration of active holes (h^+^) increases, enhancing the degradation process:

Ag^+^ + e^−^ → Ag

In addition, ultrasonic vibration induces cavitation effects, accompanied by local increases in temperature and pressure, which also promote the formation of active radicals, including ·OH and ·H. These radicals contribute further to MB degradation, providing a comprehensive mechanism involving multiple redox processes:

H_2_O (cavitation) → ·OH + ·H.

Following the scavenger tests, the electronic band-edge positions of BTNO-800 were estimated using the Mulliken electronegativity theory to clarify the origin of the observed radical selectivity and the role of piezoelectric polarization in charge dynamics.

The absolute electronegativity (χ) of Bi_3_TiNbO_9_ was calculated as the geometric mean of its constituent atoms (Bi = 4.69 eV, Ti = 3.45 eV, Nb = 4.00 eV, O = 7.54 eV), yielding χ = 6.156 eV. According to the Mulliken formalism: E_CB_ = χ − E_e_ − 2E_g_, E_VB_ = E_CB_ + E_g_, where $E_{e}=4.5$ eV is the energy of a free electron on the NHE scale, and $E_{g}=3.34$ eV is the optical band gap determined from the Tauc plot. The resulting conduction and valence band potentials of BTNO-800 are approximately E_CB_ = −0.01 eV, E_VB_ = +3.33 eV. This configuration indicates that the valence band is sufficiently positive to oxidize H_2_O or OH^−^ to hydroxyl radicals (E(H_2_O/·OH) = +1.99 eV), while the conduction band is not negative enough to reduce O_2_ to superoxide (E(O_2_/·O_2_^−^) = −0.33 eV). Consequently, ·OH radicals dominate the oxidation pathway, in full agreement with the inhibition pattern: strong suppression by IPA and EDTA-2Na, negligible effect of BQ, and acceleration by AgNO_3_, which removes conduction-band electrons and further promotes the hole-mediated oxidation cycle. Under ultrasonic vibration, mechanical deformation of the Aurivillius-type BTNO lattice generates a piezopotential that spatially separates photogenerated charges, reducing recombination and inducing band bending at the solid–liquid interface. This piezoelectric field drives holes toward the catalyst surface, facilitating ·OH generation, while electrons migrate in the opposite direction to defect sites or electron acceptors (e.g., Ag^+^). The synergistic coupling of optical excitation and piezoelectric polarization (L + U mode) therefore results in enhanced charge separation, higher ·OH yield, and a superadditive rate constant (k_L+U_ = 0.047 min^−1^ > k_L_ + k_U_ = 0.037 min^−1^).

**Figure 8:**
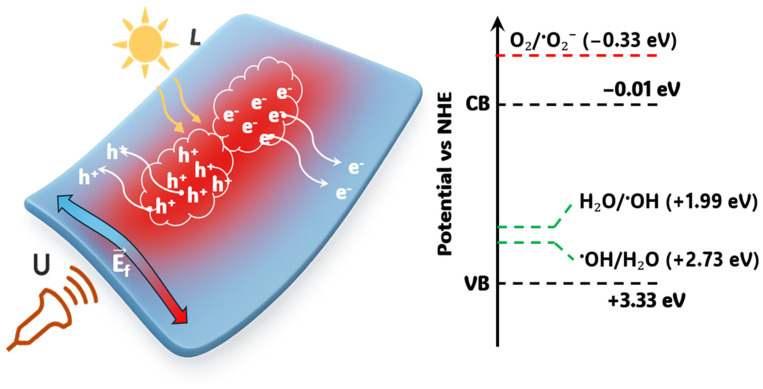
Proposed piezophotocatalytic mechanism of BTNO-800 under combined L + U. Red dotted lines indicate thermodynamically unfavorable reactions, while green dotted lines represent thermodynamically feasible reactions.

A schematic representation of the proposed piezophotocatalytic mechanism of BTNO-800 is shown in Figure 8.

### 2.6. Synergistic Effect in the Piezophotocatalisis

The obtained data indicate a fundamentally different nature of the synergistic effects in the piezophotocatalytic reactions of BTNO-900 and BTNO-800. Let us denote k_L_ as the photocatalytic rate constant, k_U_ as the piezocatalytic rate constant, and k_L+U_ as the piezophotocatalytic rate constant.

For the ceramic BTNO-900 sample, the following values were observed: k_L_ = 0.025 min^−1^, k_U_ = 0.032 min^−1^, k_L_ + k_U_ = 0.057 min^−1^. However, the experimentally determined constant for the synergistic regime was k_L+U_ = 0.049 min^−1^, which is significantly lower than the sum of the individual contributions. This subadditive effect can be attributed to overlapping reaction pathways, competition for active surface sites, and saturation of intermediate states, particularly those associated with the generation of superoxide radicals (·O_2_^−^). According to XPS analysis, the surface of BTNO-900 is characterized by a high fraction of adsorbed oxygen-hydroxyl species, which lowers the barrier for O_2_ reduction and promotes the formation of ·O_2_^−^. Furthermore, an extended carrier lifetime and a reduced bandgap were observed, both of which contribute to enhanced photocatalytic activity. Nevertheless, the excessive concentration of defects and increased photoconductivity lead to partial screening of the piezoelectric field by mobile charges, thereby diminishing the efficiency of the piezocatalytic mechanism. As a consequence, the application of mechanical stimulation does not result in a linear enhancement of the overall process but instead leads to partial duplication of the already active photocatalytic pathway, which manifests as a subadditive behavior [15,42,43].

For the melt-derived BTNO-800 sample, the kinetic constants are as follows: k_L_ = 0.011 min^−1^, k_U_ = 0.026 min^−1^, k_L_ + k_U_ = 0.037 min^−1^. In contrast, the experimentally determined value of k_L+U_ = 0.047 min^−1^, considerably exceeds the sum of the individual contributions. This superadditive effect indicates that the combined stimulation activates additional mechanisms that are inaccessible under single-mode excitation. In the case of BTNO-800, the intrinsically low photocatalytic activity is associated with the predominance of charge carrier recombination. However, ultrasonic excitation generates a piezoelectric field that spatially separates electrons and holes, effectively suppressing their recombination and thereby enabling the formation of superoxide radicals ·O_2_^−^. In the absence of defect-related conductivity, the piezoelectric field is preserved with greater stability and efficiently unlocks a previously inaccessible reaction pathway. This process accounts for the superadditive behavior, in which the observed reaction rate exceeds the simple sum of the photo- and piezo-induced contributions.

In summary, BTNO-900 and BTNO-800 exhibit contrasting scenarios of synergy: BTNO-900 demonstrates high photocatalytic activity due to abundant defects and surface oxygen-containing groups. However, these same factors increase conductivity and induce screening of the piezoelectric field, thereby limiting the contribution of the piezostimulus. The combined activation results in partial overlap of pathways and a subadditive effect. BTNO-800, in contrast, shows low intrinsic photocatalytic activity due to rapid carrier recombination. The piezoelectric field effectively mitigates this limiting factor, maintains charge separation, and activates additional pathways, leading to superadditivity.

The attempt to combine high photocatalytic activity and efficient piezocatalytic performance within a single material is associated with several fundamental contradictions arising from the different nature of charge transport and energy conversion processes. Structural, electronic, and morphological features that enhance photocatalysis often attenuate the piezocatalytic response, and vice versa.

For efficient piezocatalysis, wide-bandgap, low-conductivity materials are generally required, since under these conditions the induced piezoelectric field is maintained for longer periods and is not readily screened by mobile charge carriers. Photocatalysis, in contrast, benefits from bandgap narrowing and enhanced conductivity achieved through doping or defect engineering; however, the increase in carrier concentration accelerates the screening of polarization and reduces the efficiency of the piezoelectric effect [44].

Defects, primarily oxygen vacancies, play a decisive role. A moderate concentration of such defects promotes the adsorption of O_2_ and H_2_O, facilitates the generation of reactive radicals, and partially suppresses charge carrier recombination, thereby enhancing photocatalysis. However, an excessive concentration of vacancies increases defect-induced conductivity and creates centers for rapid recombination, which results in the screening of the piezoelectric field and a subsequent weakening of the piezocatalytic response [45,46].

Ferroelectric polarization is also of critical importance, as it generates internal electric fields and induces band bending, thereby facilitating the separation of photogenerated charge carriers. At the same time, in liquid media, surface adsorbates and ions can effectively screen this polarization, reducing both the beneficial role of the ferroelectric field in photocatalysis and the contribution of the piezoelectric effect [47]. Morphological characteristics also impose significant constraints: a high specific surface area, nanocrystallinity, and porosity enhance photocatalytic activity by increasing the number of active sites. However, excessive porosity and low mechanical strength reduce the mechanical quality factor of vibrations and, consequently, diminish the amplitude of the piezoelectric response [16]. Particular attention should be paid to the influence of illumination: the increase in the concentration of electrons and holes enhances photocatalysis but simultaneously accelerates the screening of the piezopotential, thereby limiting the contribution of mechanical activation. In contemporary literature, this compromise is described within the framework of the *piezophototronics* concept, which emphasizes the necessity of optimizing charge carrier extraction and introducing co-catalysts in order to stabilize the piezoelectric field under illumination conditions [48,49].

In Aurivillius-phase layered oxides, such as Bi_4_Ti_3_O_12_ and Bi_3_TiNbO_9_, the high spontaneous polarization is favorable for photocatalysis and photoelectrochemistry. However, the anisotropy of transport properties and the tendency toward leakage currents under defect modification significantly limit the piezocatalytic response. Contemporary approaches in this field are focused on differentiated defect control, the orientation of crystallographic domains, and the design of heterostructures, which together enable the simultaneous enhancement of both mechanisms [50,51,52].

Thus, achieving high efficiency of both photocatalysis and piezocatalysis within a single material requires a delicate balance among defect engineering, morphological design, domain structure control, and regulation of electrical conductivity. Shifting the material parameters in favor of one mechanism almost inevitably reduces the efficiency of the other, making the search for optimized composites and tailored architectures one of the key challenges in the advancement of piezophotocatalysis.

## 3. Materials and Methods

### 3.1. Sample Preparation

The samples of Bi_3_TiNbO_9_ were prepared by conventional solid-state reaction and by the molten salt method using pre-calcined analytically pure Bi_2_O_3_, Nb_2_O_5_ and TiO_2_ as starting materials. The oxides were taken in stoichiometric amounts, Bi_2_O_3_ was weighed with a 5% excess to compensate for losses during calcination.

For the synthesis of Bi_3_TiNbO_9_ by solid-state reaction, all the reactants (2 g) were mixed and ground in an agate mortar under an n-heptane layer during 1 h. The mixture obtained was dried and pelletized into tablet under the pressure of 50 bar using an Omec PI 88.00 hydraulic press (OMEC S.n.c., Certaldo, Italy). The tablets were placed into platinum crucibles with caps and calcined at 900 °C in a Nabertherm LHT 01/17 D muffle furnace.2.2 (Nabertherm GmbH, Lilienthal, Germany). This sample was labeled as BTNO-900.

For the molten salt synthesis, the same amount of metal oxides was taken with 4709 g KCl and 36,978 g NaCl. The mixture was ground in an agate mortar for an hour and pressed. The resulting mixture was placed in a platinum crucible and anneal in a muffle furnace for 2 h at 800 °C. Then the calcined product was washed with hot deionized water till the removal of the molten salt (indicating by the absence of ions Cl^−^ in water) and dried. This sample was labeled as BTNO-800.

### 3.2. Characterization

#### 3.2.1. XRD

Powder X-ray diffraction (XRD) patterns of the Bi_3_TiNbO_9_ samples were obtained using a Rigaku Miniflex II benchtop Röntgen diffractometer (Rigaku Corporation, Tokyo, Japan) with Cu K_α_ radiation in a 2*θ* angle range of 3–60° at a scanning rate of 10°/min. Phase composition of the materials was controlled using Rigaku PDXL 2.0 software and information resources of The International Centre for Diffraction Data (ICDD).

#### 3.2.2. SEM

Morphology of the samples was studied using a Zeiss Merlin scanning electron microscope (SEM) (Carl Zeiss Microscopy, Oberkochen, Germany).

#### 3.2.3. BET

The analysis of the specific surface area and porosity was carried out by nitrogen porosimetry using a Quadosorb SI device (Quantachrome Instruments, Boynton Beach, FL, USA). Before analysis, the samples were degassed under vacuum for 6 h at a temperature of 300 °C. Specific surface area was estimated according BET method, pore volume and pore diameter were calculated using DFT procedure.

#### 3.2.4. Raman Spectroscopy

Raman scattering spectra were recorded on a Bruker Senterra spectrometer (Bruker Optics Inc.,Billerica, MA, USA) in the spectral range of 100–1000 cm^−1^ using a 532 nm solid-state laser (power 0.2 mW, single accumulation time 12 s, 10 repetitions).

#### 3.2.5. XPS

X-ray photoelectron spectra (XPS) were collected on a Thermo Fisher Scientific Escalab 250Xi spectrometer (Thermo Fisher Scientific, Waltham, MA, USA) with a monochromatized Al K_α_ X-ray source (hν = 1486.6 eV) and binding energy scale calibration on a C 1s peak at 284.8 eV. The deconvolution of XPS spectra was performed using the CasaXPS software environment (Version 2.3.24PR1.0). An asymmetric Lorentzian (LA) line shape with a mixing ratio of 1.53 was applied to all components (Bi 4f, Nb 3d, Ti 2p, and O 1s) to account for possible peak distortions associated with the conductive nature of the surface and local structural defects. Background subtraction was carried out using the Shirley baseline model.

#### 3.2.6. DRS

Diffuse reflectance spectra (DRS) were measured on a Perkin Elmer Lambda 1050 spectrophotometer (PerkinElmer Inc., Waltham, MA, USA) equipped with a branded 150 mm InGaAs integrating sphere in the range of 200–2500 nm after sample deposition on a barium sulfate substrate. To find the bandgap energies E_g_, diffuse reflectance spectra of the samples were transformed into coordinates (F⋅hν)^2^ = f(hν), where F = (1 − R)^2^/2R is the Kubelka–Munk function of a reflection coefficient R. Linear sections of low-energy regions of the Kubelka–Munk graphs were extrapolated to the intersection point whose abscissa was considered as the optical bandgap energy E_g_ [53].

#### 3.2.7. TR-PLS

Time-resolved photoluminescence spectroscopy (TR-PLS) was performed using a Horiba Jobin Yvon Fluorolog-3 spectrofluorometer (HORIBA Ltd., Kyoto, Japan). The samples were excited by pulse light-emitting diode with λ_ex_ = 340 nm and the photoluminescence decays were measured at the emission maxima (λ_em_ = 410 and 405 nm for ceramic and melt-synthesized Bi_3_TiNbO_9_, respectively). To calculate average photoluminescence lifetimes τ, the luminescence decay graphs were fitted by a biexponential function I(t) = A_1_∙exp(−t/t_1_) + A_2_∙exp(−t/t_2_) + I_0_. The τ values were found via the formula τ = (A_1_∙t_1_^2^ + A_2_∙t_2_^2^)/(A_1_∙t_1_ + A_2_∙t_2_), where A_n_ and t_n_ are the amplitudes and time constants, respectively, determined during fitting [54].

### 3.3. Catalytic Studies

In this experiment, the organic dye Methylene Blue (MB) was used as a model water pollutant to evaluate the piezo-, photo-, and piezophotocatalytic properties of the synthesized Bi_3_TiNbO_9_ catalysts subjected to heat treatment at 800 °C and 900 °C. The piezocatalytic activity of the catalysts was assessed by their ability to degrade MB under ultrasonic irradiation, photocatalytic activity—under UV-visible light irradiation, and piezophotocatalytic activity—under simultaneous exposure to both light and ultrasound.

To evaluate the piezophotocatalytic activity of the Bi_3_TiNbO_9_ samples, the degradation of MB was carried out in a 2 L ultrasonic bath with a power of 120 W and a frequency of 40 kHz (Shenzhen Fanying Ultrasonic Technology Co., Ltd., Shenzhen, China), using a 150 W xenon lamp OLKs-150M (OOO “Optotekhnika”, Saint Petersburg, Russia) as the light source, without any optical filters. The experiment lasted 60 min, with absorption spectra recorded every 15 min. The experiments were conducted in a 50 mL borosilicate glass (GG17) beaker, using 20 mL of MB solution with a concentration of 2.5 mg/L and a catalyst concentration of 1 g/L. During the experiments, the temperature of the solution was maintained within 26–28 °C using a fan (for photocatalysis) and regular water replacement in the ultrasonic bath (for piezo- and piezophotocatalysis). The pH value of the solution was 7.3, corresponding to its natural value.

To confirm the role of the catalyst in the degradation of MB, control experiments were carried out under the same conditions but without the addition of the catalyst. The control experiments included exposure to ultrasound and light separately.

To determine the degradation mechanism and clarify the role of reactive oxygen species in the piezophotocatalytic process, additional experiments were performed using specific scavengers for reactive oxygen species. In particular, isopropanol was used as a hydroxyl radical (·OH) scavenger, benzoquinone for trapping superoxide radicals (·O_2_^−^), silver nitrate as an electron scavenger, and disodium salt of ethylenediaminetetraacetic acid (EDTA) to suppress hole (h^+^) activity.

## 4. Conclusions

In this work, layered Aurivillius-type Bi_3_TiNbO_9_ oxides were synthesized by two different methods—solid-state ceramic reaction at 900 °C (BTNO-900) and molten salt synthesis at 800 °C (BTNO-800)—and comprehensively investigated in terms of their structural, surface, optical, and catalytic properties. Both materials were obtained as phase-pure Aurivillius oxides, yet they exhibited pronounced differences in morphology and defect chemistry. BTNO-900 consisted of agglomerated grains with a relatively low specific surface area (1.4 m^2^/g) but possessed a higher fraction of adsorbed oxygen- and hydroxyl-related species (~57%), a slightly narrower band gap (3.21 eV), and a 2.5-fold longer average photogenerated carrier lifetime compared to BTNO-800. In contrast, BTNO-800 exhibited well-faceted, plate-like particles with a larger surface area (5.9 m^2^/g) but contained fewer oxygen-related defects (~7.2%) and showed a wider band gap (3.34 eV) together with significantly shorter carrier lifetimes.

Photocatalytic experiments demonstrated that BTNO-900 exhibited superior activity in methylene blue degradation (84.1% after 90 min, k_L_ = 0.025 min^−1^) compared to BTNO-800 (45.6%, k_L_ = 0.011 min^−1^). Under ultrasonic excitation, both samples showed comparable piezocatalytic performance (78.2% and 77.5% degradation, with k_U_ = 0.032 and 0.026 min^−1^ for BTNO-900 and BTNO-800, respectively), confirming that the piezoelectric contribution is intrinsic to the Bi_3_TiNbO_9_ structure. When light and ultrasound were applied simultaneously, both materials exhibited high overall efficiency (>92%), with apparent rate constants of k_L+U_ = 0.049 (BTNO-900) and 0.047 min^−1^ (BTNO-800). However, kinetic analysis revealed fundamentally different types of synergy. For BTNO-900, a subadditive effect was observed (k_L+U_ = 0.049 < k_L_ + k_U_ = 0.057 min^−1^), which is attributed to overlapping pathways, competition for active sites, and partial screening of the piezoelectric field by defect-induced conductivity. In contrast, BTNO-800 displayed superadditive behavior (k_L+U_ = 0.047 > k_L_ + k_U_ = 0.037 min^−1^), since the piezoelectric field effectively suppressed rapid charge carrier recombination, stabilized charge separation, and activated additional superoxide (·O_2_^−^) formation pathways that were inaccessible under isolated photocatalytic conditions.

Radical-trapping experiments confirmed hydroxyl radicals (·OH) as the dominant reactive species for both samples, while ·O_2_^−^ played a more pronounced role in BTNO-900 due to its defect-rich oxygenated surface.

Overall, BTNO-900, owing to its higher defect concentration and longer carrier lifetime, demonstrates enhanced photocatalytic activity but subadditive synergy under dual excitation, whereas BTNO-800, characterized by intrinsically low photocatalytic performance, benefits from the stabilizing effect of the piezoelectric field and exhibits superadditive synergy in piezophotocatalysis. These findings highlight the decisive role of controlled defect engineering, morphological tailoring, and conductivity optimization in balancing the multifunctional catalytic properties of Bi_3_TiNbO_9_ and related ferroelectric oxides for environmental applications.

## Figures and Tables

**Table 1:** Lattice parameters of Bi_3_TiNbO_9_ of the studied samples and data available in the literature.

	*a*, Å	*b*, Å	*c*, Å
BTNO-900	5.440	5.406	25.135
BTNO-800	5.444	5.407	25.086
Single crystal [36]	5.440	5.394	25.099
Hydrothermal-synthesized [37]	5.437	5.408	25.103

**Table 2:** Textural properties of the studied samples; S—specific surface area; V—pore volume; D—pore diameter.

	S, m^2^/g	D, nm	V, cm^3^/g
BTNO-900	1.4	2.6	0.008
BTNO-800	5.9	2.8	0.005

**Table 3:** Optical bandgap energies E_g_, long-wave edges of intrinsic light absorption λ_max_, photoluminescence maxima λ_em_ and average photoluminescence lifetimes τ at 340 nm excitation for the BTNO-800 and BTNO-900.

Synthesis Method	Intrinsic Light Absorption		Photoluminescence		
	E_g_, eV	λ_max_, nm	λ_ex_, nm	λ_em_, nm	τ, ns
BTNO-900	3.21	386.3	340	410	30
BTNO-800	3.34	371.3		405	12

## Data Availability

The raw data supporting the conclusions of this article will be made available by the authors on request.

## Supplementary Materials

The following supporting information can be downloaded at: https://www.mdpi.com/article/10.3390/molecules30204136/s1, Figure S1: SEM image of the BTNO-800 sample showing three selected regions (Spectrum 1–3) used for EDX analysis; Figure S2: SEM image of Bi_3_TiNbO_9_ particles with the sum EDX spectrum and elemental mapping of O, Na, Cl, K, Ti, Nb, and Bi; Figure S3: Nitrogen adsorption–desorption isotherms and pore size distribution curves of BTNO-800 and BTNO-900 samples; Figure S4: Linear −ln(C/C_0_) versus time plots for BTNO-800 and BTNO-900 under light, ultrasound, and combined piezophotocatalytic conditions; Figure S5: SEM images of BTNO-800 particles before and after the piezophotocatalytic process and the EDX spectrum of the analyzed region after catalysis; Table S1: Elemental composition of different regions of Bi_3_TiNbO_9_ particles determined by EDX (at.%); Table S2: Comparison of pseudo-first-order and second-order kinetic parameters for MB degradation under different activation modes for BTNO-800.
