# Supplementary material for: Tuning of Photocatalytic and Piezophotocatalytic Activity of Bi3TiNbO9 via Synthesis-Controlled Surface Defect Engineering
Source: Molecules. 2025 Oct 20;30(20):4136. doi: 10.3390/molecules30204136 (PMC12566980; doi:10.3390/molecules30204136)
Supplement: Supplementary file 1 [file molecules-30-04136-s001.zip › molecules-3921989-supplementary.pdf]

molecules

MDPI

Supplementary file

Tuning of Photocatalytic and Piezophotocatalytic Activity of Bi_3TiNbO_9 via Synthesis-Controlled Surface Defect Engineering

Farid F. Orudzhev [a,b*], Asiyat G. Magomedova [a], Sergei A. Kurnosenko [c], Vladislav E. Beklemyshev [c], Wei Li [d], Chuanyi Wang [e] and Irina A. Zvereva [c*]

[a] Smart Materials Laboratory, Dagestan State University, Makhachkala, St. M. Gadjieva 43-a, Dagestan Republic 367000, Russia
[b] Geothermal and Renewal Energy Institute of the Russian Academy of Sciences, Makhachkala, Ave. 39-a, Dagestan Republic 367030, Russia
[c] Department of Chemical Thermodynamics and Kinetics, Saint Petersburg State University, 7/9 Universitetskaya nab., St. Petersburg, 199034, Russia
[d] School of Chemistry and Chemical Engineering, Shaanxi University of Science and Technology, Xi'an 710021, PR China
[e] School of Environmental Science and Engineering, Shaanxi University of Science and Technology, Xi'an 710021, PR China
* Correspondence: farid-stkha@mail.ru (F.F.O.); irina.zvereva@spbu.ru (I.A.Z.)

Figure S1. SEM image of the BTNO-800 sample showing three selected regions (Spectrum 1–3) used for EDX analysis.

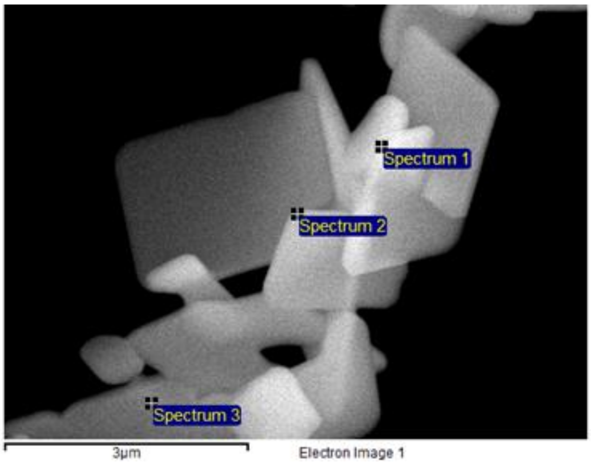

Table S1. Elemental composition of different regions of Bi_3TiNbO_9 particles determined by EDX (at.%).

Analysis area	O (at.%)	Na (at.%)	Cl (at.%)	K (at.%)	Ti (at.%)	Nb (at.%)	Bi (at.%)	Σ = 100%
Spectrum 1	17.97	0.44	0.25	—	4.16	11.15	66.03	100
Spectrum 2	21.55	—	—	—	3.69	10.51	64.25	100
Spectrum 3	7.83	—	—	—	5.05	14.96	72.15	100
Sum Spectrum	13.34	0.30	0.21	—	4.76	12.04	69.35	100

Analysis of three local regions (Spectrum 1–3, see Fig. S1, Table S1) confirmed the presence of Bi, Ti, Nb, and O elements corresponding to the expected composition of the Bi_3TiNbO_9 phase. Minor impurities of Na, Cl, and K (<0.5 at.%) are attributed to residual precursor salts. The Bi content varies from 64 to 72 at.%, indicating a slight heterogeneity in the distribution of the heavy element on the surface of agglomerates. The averaged values from the sum spectrum (Bi ≈ 69 at.%, Nb ≈ 12 at.%, Ti ≈ 5 at.%) are in good agreement with the stoichiometric ratios calculated for Bi_3TiNbO_9.

Figure S2. SEM image of Bi_3TiNbO_9 particles (upper left), the sum EDX spectrum (bottom left), and elemental mapping of O, Na, Cl, K, Ti, Nb, and Bi (right).

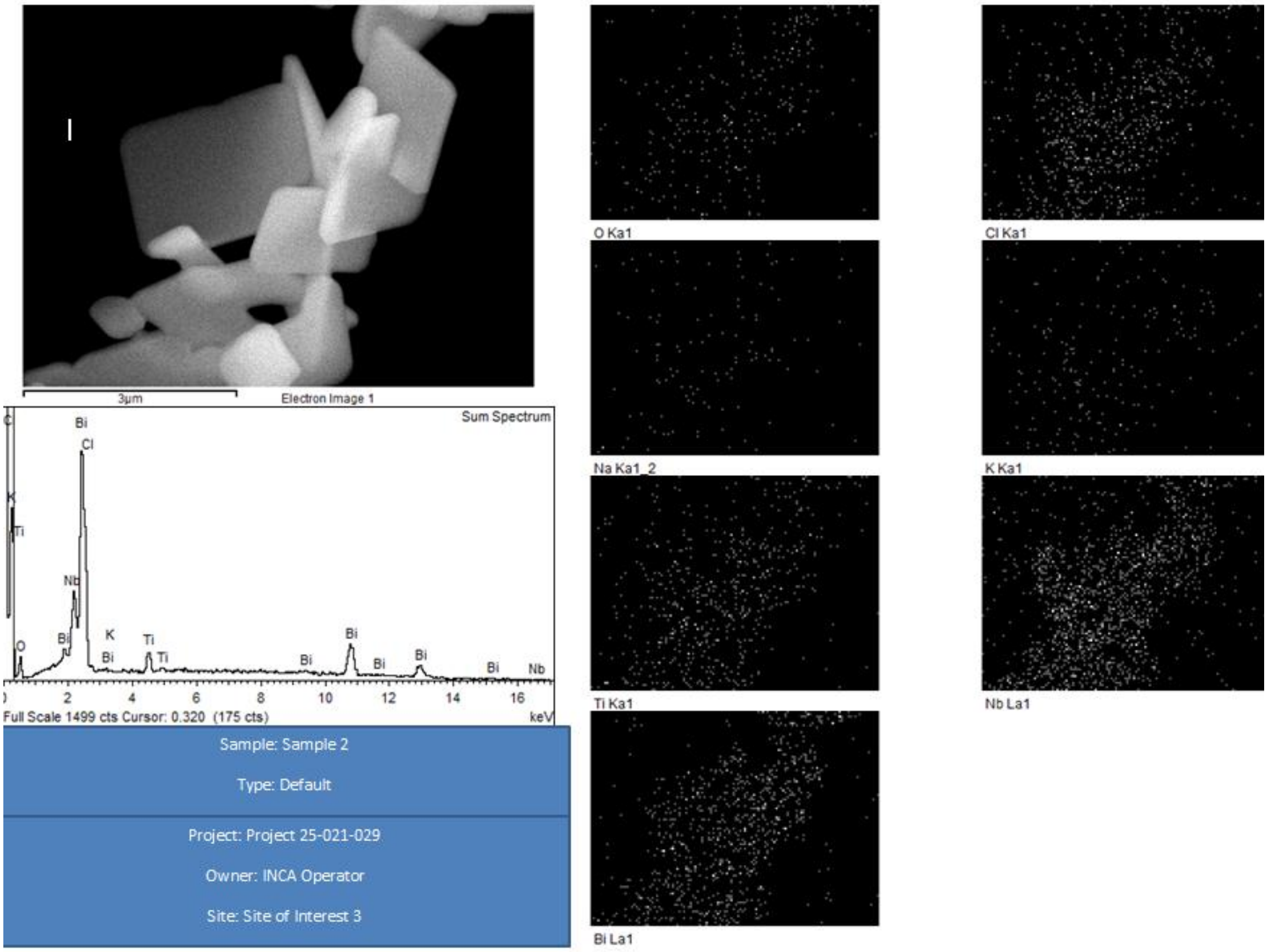

Elemental mapping confirms the homogeneous distribution of the main elements Bi, Ti, Nb, and O within each particle, indicating the formation of a single-phase Bi_3TiNbO_9 structure (Fig. S4). Signals of Na, Cl, and K appear locally and exhibit low intensity, which correlates with their low concentration (<0.5 at.%) obtained from quantitative analysis (see Table S1). These minor impurities are likely associated with residual precursor salts or adsorbed species. The sum spectrum clearly shows characteristic peaks of Bi, Nb, Ti, and O, confirming the presence of all components of the perovskite-like phase. The absence of any additional peaks, except for weak impurity lines, demonstrates the high purity of the obtained material.

Figure S3. Nitrogen adsorption–desorption isotherms (top) and pore size distribution curves (bottom) of BTNO-800 and BTNO-900 samples.

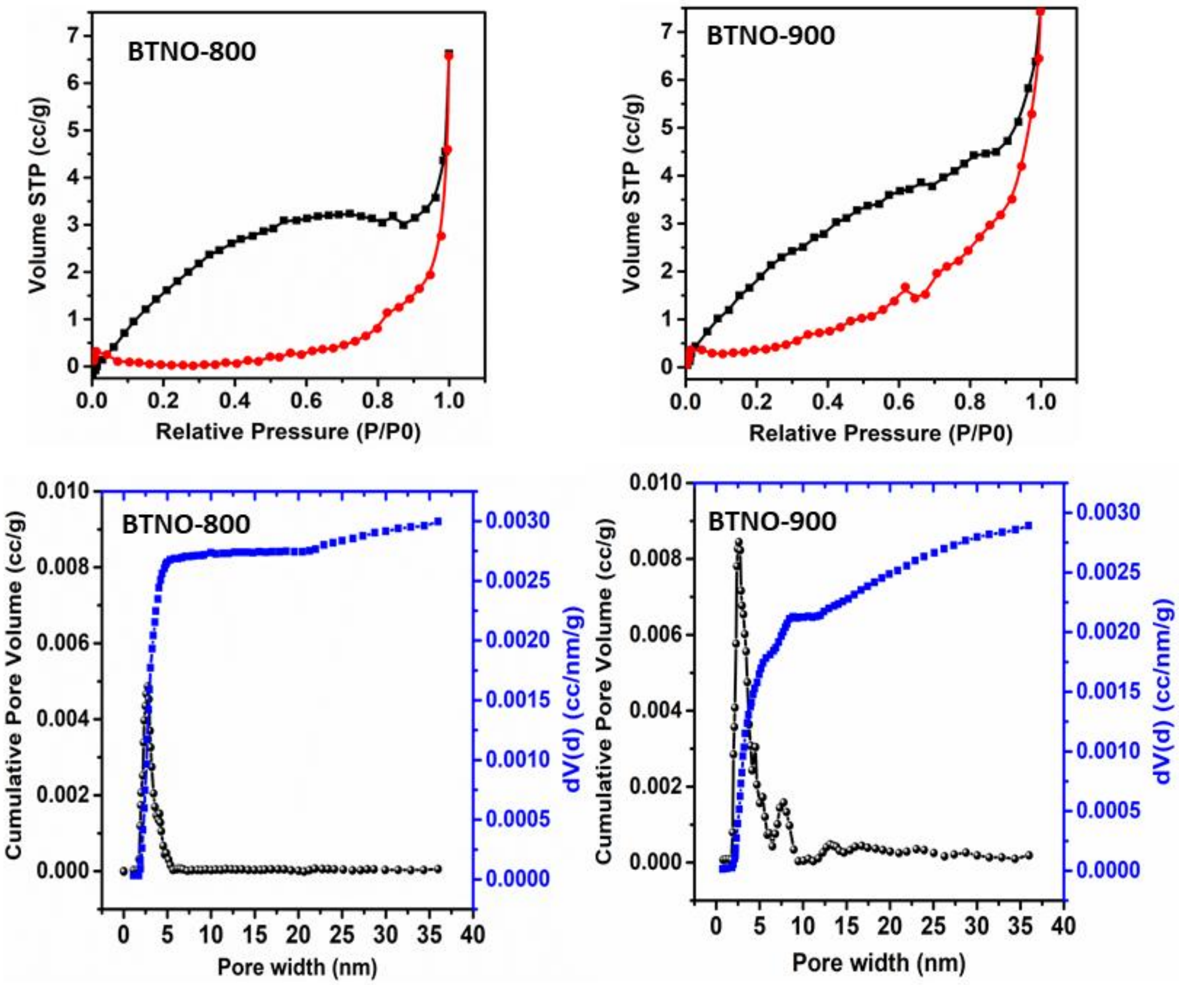

Figure S4. Linear −ln(C/C$_0$) vs time plots for BTNO-800 (a) and BTNO-900 (b) under *Light*, *US*, and *Combined* regimes. Solid lines denote linear fits of the pseudo-first-order Langmuir–Hinshelwood model; k ± SE (min^{-1}) and R^2 are reported on the insert.

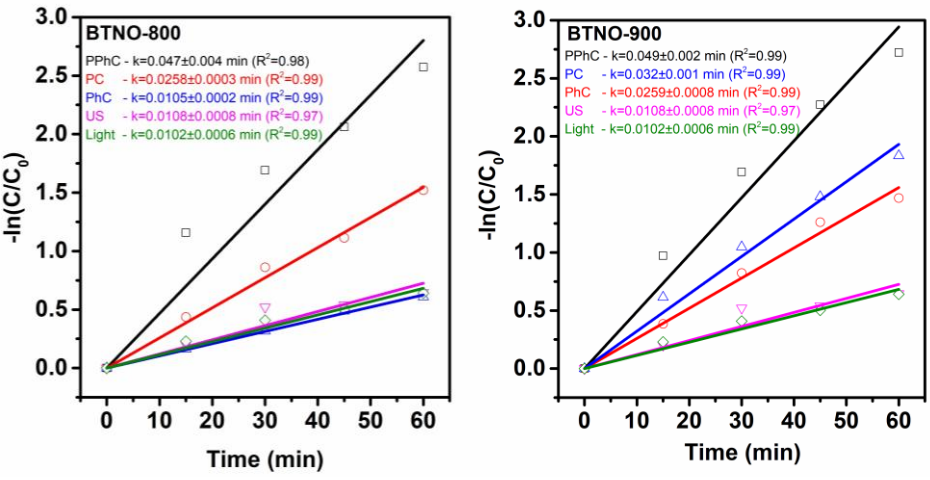

To verify the correctness of the selected kinetic model for methylene blue (MB) degradation, the dependencies calculated using the pseudo-first-order (Langmuir–Hinshelwood type) and second-order equations were compared. The corresponding rate constants (k_1 and k_2) and correlation coefficients (R^2) are summarized in Table S2.

Table S2. Comparison of pseudo-first-order and second-order kinetic parameters for MB degradation under different activation modes for BTNO-800.

	**Pseudo-first-order**		**Second-order**	
**Process**	**k_1 (min^{-1})**	**$R^2_{(1)}$**	**k_2 ($L·mg^{-1}·min^{-1}$)**	**$R^2_{(2)}$**
**PPhC**	0.047	0.98	0.071	0.95
**PC**	0.0258	0.99	0.021	0.95
**PhC**	0.0105	0.99	0.0055	0.99
**US**	0.0108	0.97	0.0065	0.92
**Light**	0.0102	0.99	0.0061	0.99

Analysis of the data shows that, for all studied processes (photolysis, sonolysis, photocatalysis, piezocatalysis, and piezophotocatalysis), the pseudo-first-order model exhibits slightly higher or comparable R^2 values (0.97–0.99) compared to the second-order model (0.92–0.99). The advantage of the first-order model is particularly evident for piezocatalytic and piezophotocatalytic reactions, indicating its better agreement with the experimental data. Such behavior is typical for heterogeneous catalytic reactions at low initial concentrations of the organic substrate, where the reaction rate is governed by surface-controlled stages — the generation and interfacial transfer of reactive radical species (·OH, $·O_2^-$). It should also be noted that, prior to irradiation or ultrasonic activation, the suspensions were kept in the dark for 30 minutes to reach adsorption–desorption equilibrium, ensuring an approximately constant surface coverage of active sites throughout the reaction.

Therefore, the application of the pseudo-first-order (Langmuir–Hinshelwood) model is physically and kinetically justified for describing MB degradation in the examined systems. The use of the second-order model does not improve the fitting quality and does not alter the obtained conclusions regarding the mechanism and the synergistic effect of the combined piezo- and photocatalytic processes. The enhanced degradation rate observed in the PPhC (piezophotocatalytic) mode compared to PC and PhC confirms that the primary origin of the synergy lies not in the change of reaction order, but in the efficient separation of photoinduced charge carriers under the influence of the piezoelectric field within the active material.

Figure S5. (A) SEM image of BTNO-800 particles before the piezophotocatalytic process; (B) after the process; (C) EDX spectrum of the analyzed region after catalysis.

A

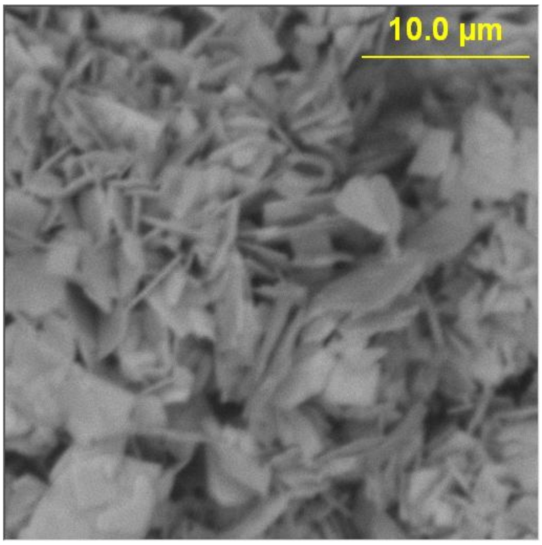

B

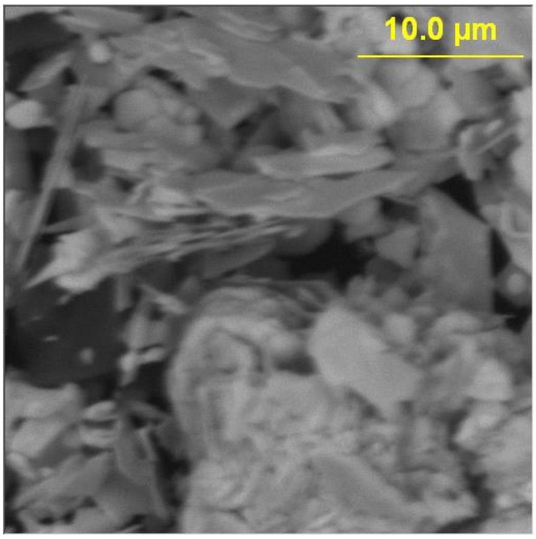

C

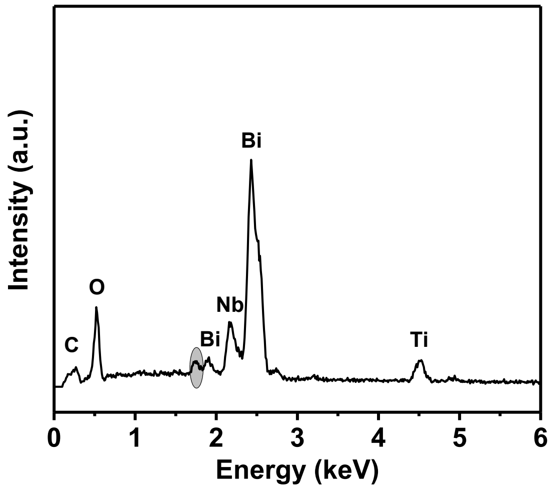

The SEM image of the pristine powder (Fig. S5A) shows well-crystallized plate-like particles. After the piezophotocatalytic process (Fig. S5B), the overall morphology of the particles remains unchanged: the shape and size of the crystallites are preserved, indicating high mechanical and structural stability of the material under cyclic loading. Particular attention should be paid to the EDX results (Fig. S5C). In the pristine BTNO-800 sample, weak signals of Na, K, and Cl were detected, which are associated with residual precursor salts and slightly adsorbed ionic species on the particle surface. After the piezophotocatalytic process, these elements completely disappear from the spectrum, indicating efficient removal of surface contaminants and a self-cleaning effect during catalysis. The main elements Bi, Ti, Nb, and O remain stable, and their relative ratios correspond to the stoichiometric composition of Bi_3TiNbO_9.

It is noteworthy that a weak signal appears in the region corresponding to silicon (Si, highlighted area). This peak is most likely related to the interaction of the catalyst particles with the glass surface of the reaction vessel during the experiment. The appearance of Si does not indicate its chemical incorporation into the structure but rather superficial contamination caused by contact between the particles and the glass walls under cavitation conditions.
